# Relationship Between Polycyclic Aromatic Hydrocarbons and Cardiovascular Diseases: A Systematic Review

**DOI:** 10.3389/fpubh.2021.763706

**Published:** 2021-12-07

**Authors:** Manthar Ali Mallah, Mukhtiar Ali Mallah, Yang Liu, He Xi, Wei Wang, Feifei Feng, Qiao Zhang

**Affiliations:** ^1^Department of Toxicology, College of Public Health, Zhengzhou University, Zhengzhou, China; ^2^Department of Chemical Engineering, Quaid-e-Awam University of Engineering, Science & Technology (QUEST), Nawabshah, Pakistan; ^3^Department of Occupational and Environmental Health, College of Public Health, Zhengzhou University, Zhengzhou, China

**Keywords:** cardiovascular diseases, hypertension, heart rate variability, polycyclic aromatic hydrocarbons, PAH exposure

## Abstract

**Objective:** The primary aim of this systematic review was to examine the relationship of polycyclic aromatic hydrocarbon (PAH) exposure with cardiovascular diseases (CVDs) and elaborate the current knowledge and recent advances in the area of PAH and its effects on CVDs and discuss the growing epidemiological evidence linking PAH to CVDs on the health of human populations. In this systematic review, the increased risk of cardiovascular diseases and their relationship with PAHs were discussed in detail.

**Methods:** On 05th April 2021, a systematic literature search was conducted using PubMed/Medline and Web of Science search engines in accordance with the Preferred Reporting Items for Systematic Reviews and Meta-Analyses (PRISMA) criteria. The search was limited to articles that were written in English and dealt with human issues. All original peer-review publications were considered for inclusion. Comments, case reports, reviews, duplicated papers, and conference reports were excluded. Data was collected from included papers by two independent reviewers.

**Results:** Conclusively, 20 research articles published between 2005 and 2021 were chosen for the final analysis. The systemic review included 20 studies with a variety of geographical studies. The most common research category among the nominated studies were time-series studies followed by retrospective cohort, cross-sectional, quasi-experimental, panel, and case-control studies. Most of the studies were conducted in the United States, whereas others were showed in various geographical countries around the world, such as Denmark, Germany, Finland, Netherlands, France, China, Norway, Korea, Sweden, Saudi Arabia, and Belgium. Eight studies assessed the association between PAH exposure and CVDs, four articles observed this relationship with blood pressure (BP), two observed association between atherosclerotic CVD and PAH, one congenital heart disease, cardiovascular events, and two with obesity. Furthermore, in some investigations, a favorable association between PAH exposure and hypertension as well as PAH exposure and obesity was found.

**Conclusion:** In conclusion, this systematic review examined the relationship of PAH exposure with CVDs and CVD-related risk factors by searching several digital databases. After a comprehensive literature searches and summarizing findings from 20 articles, the authors concluded that a positive relationship was observed between PAH exposure and CVD risks.

**Graphical Abstract:**
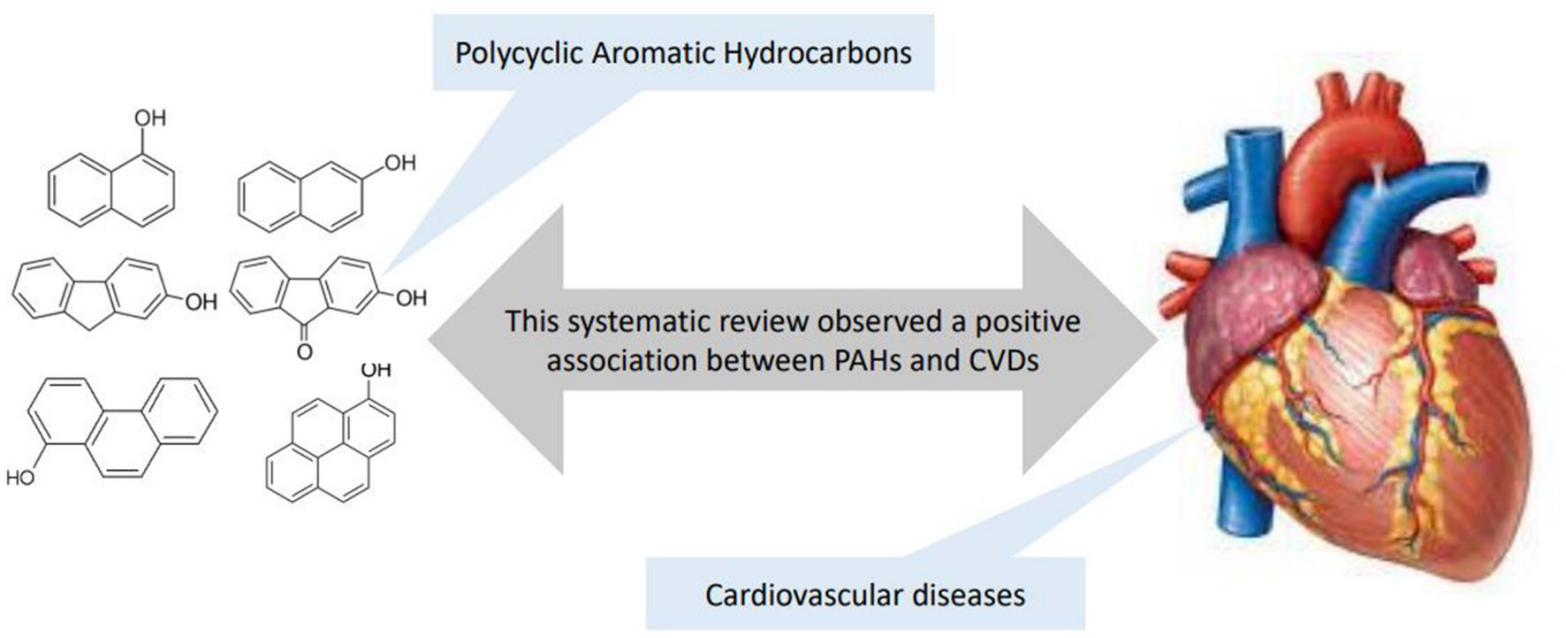
This figure shows relationship between PAHs and CVDs.

## Introduction

Polycyclic aromatic hydrocarbons (PAHs) are made up of two or more fused benzene rings. These ubiquitous contaminants are formed naturally or by incomplete combustion or through organic matter (1). Furthermore, to the well-documented oncogenic impacts of PAH (2), PAH exposure in the surrounding environment and at work can lead to the growth of CVDs, such as peripheral arterial disease (PAD), coronary heart disease (CAD), myocardial infarction (MI), and stroke (3–7). In the U.S. general population, for example, urinary PAH biomarkers, such as 2-OH-PH are associated with raised CVD events (median = 0.061 μg/L for the 2001–2002 and 2003-2004 surveys, correspondingly) (5). In addition, raised prevalence of fatal ischemic heart disease in relation to work-related exposure to benzo(a)pyrene B[a]P (*n* = 12,367 males asphalt pavers from 7 nations) was identified in earlier research of asphalt pavers (3). The underlying mechanisms are not known, but it has been advised that oxidative stress and systemic inflammation perform a role in CVD caused by PAH (8, 9). Moreover, it is predicted that particle matter in the ambient air population caused around 3 million fatalities and 85 million disability-adjusted live years (DALYs) in 2012 (10). In most parts of the world, ambient air pollution has been on the rise rapidly due to industrialization, urbanization, and motorization (10). Ambient air contaminants in the atmosphere consist of particulate matter (PM), organic compounds, gases, and toxic metals (11). Liquid droplets (aerosols) and solid particles such as dirt, dust, smoke, and soot are absorbed by the PM. The PM is a kind of particle which is detected in smoke, exhaust fumes, and smog that is produced by combustion or as a consequence of a reaction to gases, sunlight, or air (12). As demonstrated in the literature, various factors such as poor diet, stress, and environmental contaminant exposure (13), might CVDs caused by atherosclerosis, myocardial infarction, and angina pectoris (14). Epidemiological studies showed that exposure to definite substances in the air can cause increased CVDs risk in human individuals (15). A major component of air is PAHs, which are positively correlated with cardio-metabolic risk factors and atherosclerosis (8, 9, 16).

The body's natural response to damage is inflammation, which includes vascular hyper-permeability, white cell proliferation, and vascular remodeling. Exposure to PAHs was found to be favorably linked with an inflammatory response in both *in vivo* and *in vitro* investigations (15, 17). Furthermore, intrinsic and extrinsic factors (including angiotensin-II and air pollution) can cause oxidative stress and activation of inflammatory cytokines (like IL-1β and TNF-α), resulting in endothelial dysfunction, smooth muscle cell relation reduction, and carotid artery intima-media thickening. Though endothelial dysfunction may have a role in the onset of blood vessels inflammation and the formation of vessels remodeling, it has also been shown to exacerbate plaque formation and instability in atherosclerosis (18). As a result, inflammation is regarded as an initial stage in the development of atherosclerosis.

PAH exposure is known to be associated with a decrease in cardiac autonomic function (19). In addition, PAH exposures have been documented to be positively associated in certain occupational circumstances with CVD-caused mortality (3, 20). In addition, exposure to PAH has been shown to aggravate atherosclerosis via inflammation (21). The primary aim of this systematic review was to examine the relationship of polycyclic aromatic hydrocarbon (PAH) exposure with cardiovascular diseases (CVDs) and elaborate the current knowledge and recent advances in the area of PAH and its effects on CVDs and discuss the growing epidemiological evidence linking PAH to CVDs on the health of human populations. In this systematic review, the increased risk of cardiovascular diseases and their relationship with PAHs were discussed in detail.

## Methods

### Search Strategy and Selection Criteria

**Figure 1:**
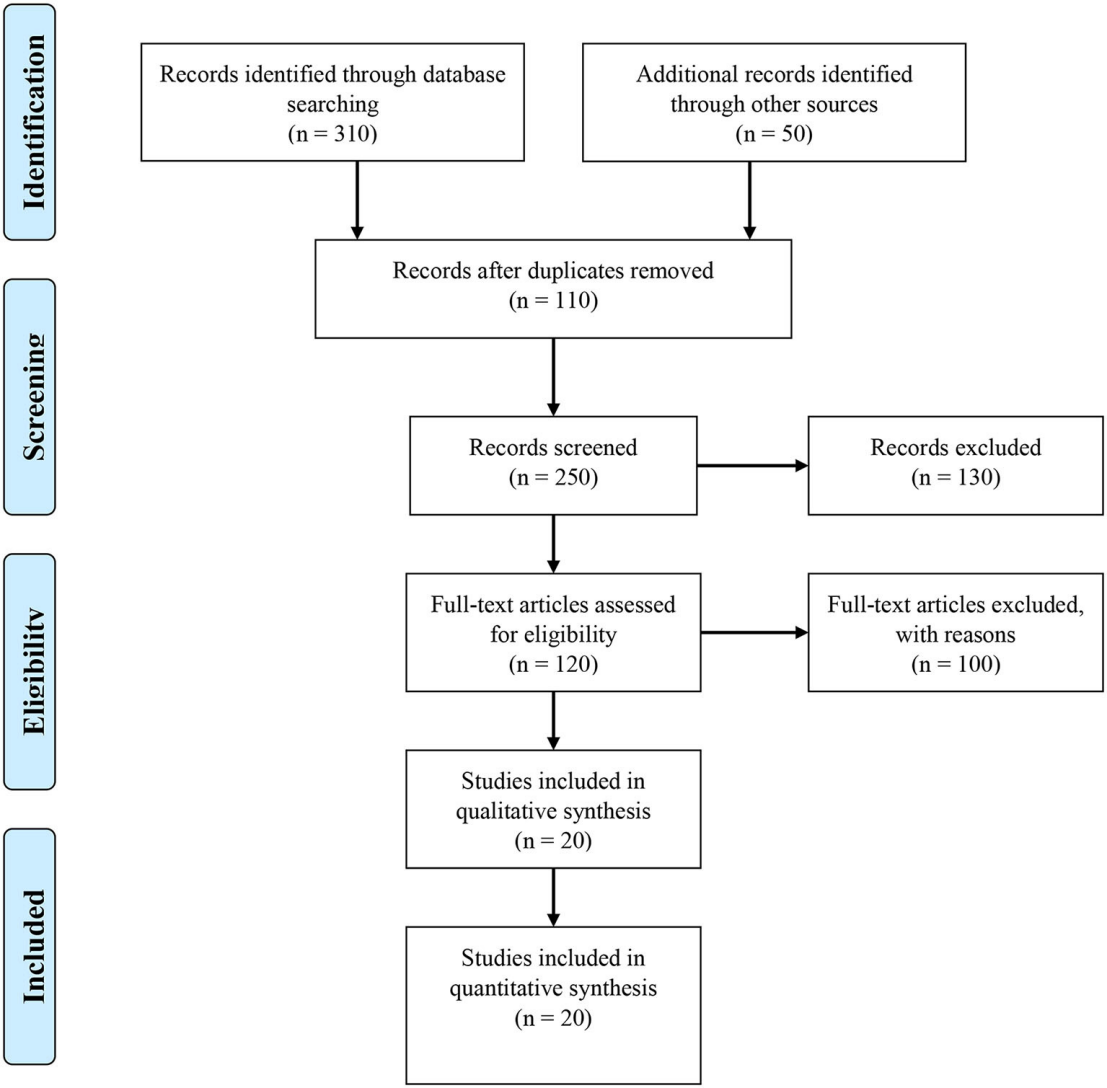
Identification, screening, eligibility and inclusion of studies for the systematic review.

On 05th April 2021, a systematic literature search was conducted using PubMed/Medline, and Web of Science search engines in accordance with the Preferred Reporting Items for Systematic Reviews and Meta-Analyses (PRISMA) criteria (22). The study enrollment method was described using a Preferred Reporting Items for Systemic Review and Meta-Analysis (PRISMA) 2009 flow diagram (Figure 1). In our search approach, we utilized MeSH keywords and Emtree terms, as well as other relevant free-text terms. The keywords used for search were “PAHs,” “polycyclic aromatic hydrocarbons,” “polycyclic aromatic compounds,” “particulate matter,” combined with “cardiovascular diseases,” “cardio-metabolic disorders,” “hypertension,” and “cardiovascular disease risk.” Search strategy is detailed in Supplementary Table 1.

The search was limited to articles that were written in English and dealt with human issues. The inclusion criteria were followed based on: papers that focused on PAHs and cardiovascular diseases, an association of urinary PAHs and hypertension, exposure of PAHs and cardiovascular diseases, and association of cardiovascular risk factors and PAHs. We carefully examined the bibliographies of the papers that were included in order to find any relevant studies that were missed by the first search. All original peer-review publications were considered for inclusion. When more than one report has almost the same material, the most recent research publications were chosen. Comments, case reports, reviews, duplicated papers, and conference reports were excluded.

### Data Extraction and Quality Assessment

Data was collected from included papers by two independent reviewers (SB and MS). The author's name, publication year, study design, study participants, geographical locations, importance, and key findings were all extracted using Microsoft Excel. Newcastle-Ottawa scale for cohort studies and the modified Newcastle-Ottawa scale for cross-sectional studies were used to assess the quality of the included research, both of which have been validated and widely used in prior studies (23, 24). These studies were assessed using these scales by two independent reviewers (SB & MS), and any disagreements were addressed through discussion and the average score was utilized. A consensus has been made if there were any disagreements between the two reviewers concerning the methodological quality of an article. We used a practical technique to choose the majority of possible literature when data from the same study was provided in two or more publications.

## Results

### Study Selection and General Characteristics

Figure 1 shows a flowchart of the publication selection technique. In the first stage, a total 360 studies were collected from various internet sources. The remaining 250 studies were reviewed based on title and abstract, with 110 being duplicated articles that were eliminated. A total of 130 reports were eliminated, including 100 irrelevant studies and 30 duplicates. 120 studies were evaluated for eligibility and 100 articles were eliminated based on inadequate data (*n* = 80), irrelevant studies (*n* = 15), and duplicate (*n* = 5). Conclusively, 20 research articles published between 2005 and 2021 were chosen for the final analysis. All of the studies that were chosen were written in English. The characteristics of the studies are detailed in Table 1.

**Table 1:** Characteristics (summary) of included studies in the systematic review.

**Source**	**Study title**	**Study design**	**Sample size**	**Setting**	**Implication**	**Exposure assessment**	**Key findings**	**Quality score**
Cao et al. (25)	Polycyclic aromatic hydrocarbon exposure and atherosclerotic cardiovascular disease risk in urban adults	Cohort study	3,052 participants	Wuhan, China	ASCVD	Gas chromatography coupled with mass spectrometry (Agilent 6890N/59758B, Agilent Technologies Inc. CA) was used to measure urinary concentrations of twelve OH-PAHs, which contains three high molecular weight OH-PAHs [3-hydroxybenzo[a]pyrene, 6-hydroxychrysene and 1-hydroxypyrene (1-OHP)] and nine low molecular weight OH-PAHs (1-, 2- hydroxynaphthalene, 2-, 9- hydroxyfluorene, and 1-, 2-, 3-, 4-, 9- hydroxyphenanthrene).	Significantly positive dose-response relationships between total urinary hydroxynaphthalene (∑OHNa), hydroxyfluorene (∑OHFlu), hydroxyphenanthrene (∑OHPh), and 10-year ASCVD risk were observed (all *P* for trend <0.05). Positive relationships between urinary OH-PAH levels and 8-oxodG, as well as between urinary 8-oxod G levels and 10-year risk of ASCVD. Mediation analyses indicated that urinary 8-oxodG mediated 14.49, 12.62, and 10.55% of the associations between urinary ∑LMW OH-PAHs, ∑OHNa, ∑OHFlu, and 10-year ASCVD risk, respectively. The oxidatively damaged DNA pathway may be a possible mechanism underlying PAH-associated ASCVD risk elevation	9
Lee et al. (26)	Association between urinary polycyclic aromatic hydrocarbons and hypertension in the Korean population	Cross-sectional study	6,478 adults	Korea	Hypertension	Urinary PAH metabolite concentrations were analyzed by gas chromatography-mass spectrometry (GC–MS). Exposure to PAHs was calculated with concentrations of four urinary PAH metabolites: 2-naphthol, 1-hydroxyphenanthrene, 2-hydroxyfuorene, and 1-hydroxypyrene. Each urinary PAH metabolite was categorized by quartile: quartile 1 (≤ 25th percentile) vs. quartile 2 (>25th and ≤ 50th percentile) vs. quartile 3 (>50th and ≤ 75th percentile) vs. quartile 4 (>75th percentile).	Urinary 1-hydroxyphenanthrene was significantly higher in the hypertension group than in the non-hypertension group. In 1-hydroxyphenanthrene, the OR for hypertension was significantly higher in the third and fourth quartile groups than in the first quartile group (third: OR 1.707, 95% CI 1.203–2.423, fourth: OR 1.604, 95% CI 1.158–2.223). Results suggest an association between exposure to PAHs and hypertension in a Korean adult population	10
Yang et al. (27)	Heart rate variability mediates the association between polycyclic aromatic hydrocarbons exposure and atherosclerotic cardiovascular disease risk in coke oven workers	Cross-sectional study	1,628 Coke oven workers	Wuhan, China	ASCVD, HRV	The urinary levels of 12 PAH metabolites [1-hydroxynaphthalene (1-OHNa); 2-hydroxynaphthalene (2-OHNa); 2-hydroxyfluorene (2-OHFlu); 9-hydroxyfluorene (9-OHFlu); 1-hydroxyphenanthrene (1-OHPh); 2-hydroxyphenanthrene (2-OHPh); 3-hydroxyphenanthrene (3-OHPh); 4-hydroxyphenanthrene (4-OHPh); 9-Hydroxyphenanthrene (9-OHPh); 1-hydroxypyrene (1-OHP); 6-hydroxychrysene (6-OHChr); 3-hydroxybenzo[a]pyrene (3-OHBaP)] were determined by gas chromatography-mass spectrometry (GC-MS, Agilent, Santa Clara, CA).	Elevated PAHs metabolites were dose-responsive related to increased risk of ASCVD among coke oven workers. Urinary 1-hydroxynaphthalene (1-OHNa), 2-OHNa, and total PAH metabolites (ΣOH-PAH) were dose-responsive associated with an increased risk of ASCVD. Compared with the lowest quartile, the adjusted odds ratio (OR) for ASCVD risk in the highest quartile were 2.36 for 1-OHNa, 6.58 for 2-OHNa, and 1.60 for ΣOH-PAH (all Ptrend < 0.05). Mediation analyses indicated that HRV mediates 2.7%-4.3% of the association between 2-OHNa exposure and higher ASCVD risk.	09
Hu et al. (28)	Association of polycyclic aromatic hydrocarbons exposure with atherosclerotic cardiovascular disease risk	Cohort study	2,020 subjects (689 men and 1,333 women)	Wuhan, China	ASCVD	Urinary OH-PAHs were measured by a gas chromatography-mass spectrometry, ten urinary OHPAHs (including 1-hydroxynaphthalene, 2-hydroxynaphthalene, 2-hydroxyfluorene, 9-hydroxyfluorene, 1-hydroxyphenanthrene, 2-hydroxyphenanthrene, 3-hydroxyphenanthrene, 4- hydroxyphenanthrene, 9-hydroxyphenanthrene and 1- Hydroxypyrene) were detected.	Some OH-PAHs were positively associated with ASCVD risk but not CHD risk, including 2-hydroxyfluoren (β = 1.761; 95% CI: 1.194–2.597), 9-hydroxyfluoren (β = 1.470; 95% CI: 1.139–1.898), 1-hydroxyphenanthrene (β = 1.480; 95% CI: 1.008–2.175) and ΣOH-PAHs levels (β = 1.699; 95% CI: 1.151–2.507). Exposure to PAHs may increase the risk of atherosclerosis, which was partially mediated by MPV or CC16	08
Li et al. (29)	Assessment of interaction between maternal polycyclic aromatic hydrocarbons exposure and genetic polymorphisms on the risk of congenital heart diseases	Case-control study	357 mothers of CHDs fetuses and 270 control mothers	China	CHDs	1-Hydroxypyrene-glucuronide (1-OHPG) is a persistent PAH metabolite excreted in the urine that serves as an index biomarker for mixed PAH exposure.	The higher-level PAHs exposure was associated with the risk of CHDs (aOR = 2.029, 95% CI: 1.266, 3.251). The multiplicative-scale interactions between PAHs exposure and polymorphisms of CYP1A2 rs4646425 (*P* = 0.03) or CYP2E1 rs915908 (P = 0.0238) on the risk of CHDs were observed. This study suggests that maternal AHR polymorphisms may modify the association of PAHs exposure with CHDs, CYP1A2 or CYP2E1 polymorphisms significantly interact with PAHs exposure on CHDs.	10
Yin et al. (30)	Obesity mediated the association of exposure to polycyclic aromatic hydrocarbon with the risk of cardiovascular events	Cross-sectional study	1,240 residents	China	Cardiovascular events	Eight urinary OH-PAHs [including 2-hydroxynaphthalene (2-OHNa), 2- hydroxyfluorene (2-OHFlu), 3-hydroxyfluorene (3-OHFlu), 2- hydroxyphenanthrene (2-OHPh), 3-hydroxyphenanthrene (3-OHPh), 4-hydroxyphenanthrene (4-OHPh), 9-hydroxyphenanthrene (9- OHPh), 1-hydroxypyrene (1-OHP)] were measured from urine samples.	The positive association between urinary PAHs metabolites and BP or the odds ratios for high BP (all *P* <0.05). Each one-unit increase in ln-transformed urinary levels of 4-hydroxyphenanthrene or the total of PAH metabolites was associated with a 12.63% or 11.91% increase in the estimated 10-year ASCVD risk (both *P* < 0.05). The associations between elevated levels of ∑OH-PAH or PAH individuals and high BP, whereas the association of urinary level of ∑OH-PAH or 4- OHPh with increased risks for ASCVD PAHs exposure may be associated with elevated BP and an increased risk of ASCVD	09
Alhamdow et al. (31)	Early markers of cardiovascular disease are associated with occupational exposure to polycyclic aromatic hydrocarbons	Cross-sectional study	151 chimney sweeps and 152 controls males	Sweden	CVD	The PAH metabolites in urine were measured using liquid chromatography coupled to tandem mass spectrometry (LC-MS/MS; QTRAP 5500, AB Sciex, Foster City, CA, USA). 1-hydroxypyrene (1-OH-PYR) is a pyrene metabolite that has been widely utilized as a proxy for total PAH exposure. The metabolite 3-hydroxybenzo[a]pyrene (3-OH-BaP) was tested for exposure to benzo[a]pyrene; the	Compared with controls, chimney sweeps had increased homocysteine, cholesterol, and HDL (β = 3.4 μmol/L, 0.43 mmol/L, and 0.13 mmol/L, respectively, *P* ≤ 0.003, adjusted for age, BMI, and smoking). In chimney sweeps, PAH metabolites correlated positively with the percentage of soot sweeping (*P* < 0.001). 2-hydroxyphenanthrene, 3-hydroxybenzo[a]pyrene, and	08
						metabolites 2-hydroxyphenanthrene (2-OH-PH) and 3-hydroxybenzo[a]anthracene (3-OH-BaA) were assessed for exposure to phenanthrene and benzo[a]anthracene, respectively.	3-hydroxybenzo[a]anthracene were positively associated with diastolic BP (*P* <0.044, adjusted for age, BMI, and smoking). PAH exposure among chimney sweeps resulted in elevated levels of markers for CVD risk.	
Yang et al. (32)	Effects of coke oven emissions and benzo[a]pyrene on blood pressure and electrocardiogram in coke oven workers	Cross-sectional Study	880 coke oven workers and 710 oxygen employees	Chongqing, China	Hypertension and ECG	Measurement of Benzo [a] pyrene concentration High-performance liquid chromatography (HPLC; 1100, Agilent, Palo Alto, CA) was performed to analyze airborne B[a]P concentrations.	Age and BP[a] exposure were risk factors for hypertension in coke oven workers (*P*> 0.005) and both were risk factors for abnormal ECG (*P* < 0.005). BP[a] and COE exposures are risk factors for hypertension and abnormal ECG in coke oven workers.	10
Alshaarawy et al. (33)	The association of urinary polycyclic aromatic hydrocarbon biomarkers and cardiovascular disease in the US population	Retrospective cohort 2001–2010	3,550 males and 3,751 females	US	Cardiovascular disease	Gas chromatography combined with high resolution mass spectrometry (GCHRMS) was used to measure PAH analytes. Seven urinary low molecular weight PAH analytes (1-hydroxynapthol, 2-hydroxynapthol, 2- hydroxyfluorene, 3-hyrdroxyfluorene, 1-hydroxyphenanthrene, 2-hydroxyphenanthrene, 3- hydroxyphenanthrene) and one urinary high molecular weight analyte (1-hydroxypyrene) were measured.	PAH exposure was positively associated with CVD (β = 0.12; 95% CI: 0.03–0.20)	09
Yang et al. (34)	Exposure to Polycyclic Aromatic Hydrocarbons, Plasma Cytokines, and Heart Rate Variability	Quasi-experimental	489 coke-oven workers	US	HRV	Gas chromatography-mass spectrometry used to measure the concentrations of 12 urinary OH-PAHs: 10 were non-carcinogenic metabolites (1-hydroxypyrene, 1-hydroxynaphthalene, 2-hydroxynaphthalene, 2-hydroxyfluorene, 9-hydroxyfluorene, 1-hydroxyphenanthrene, 2-hydroxyphenanthrene, 3-hydroxyphenanthrene, 4-hydroxyphenanthrene, and 9-hydroxyphenanthrene), and 2 were carcinogenic metabolites (6-hydroxychrysene and 3-hydroxybenzo[a]pyrene).	PAH exposure was associated with plasma cytokines were associated with decreased HRV (*P* < 0.05)	09
Feng et al. (19)	A community study of the effect of polycyclic aromatic hydrocarbon metabolites on heart rate variability based on the Framingham risk score	Cross-sectional	1,978 adult residents	Wuhan, China	HRV	12 urinary PAH metabolites [pyrene metabolite: 1-OHP; naphthalene metabolites: 1-hydroxynaphthalene (1-OHNa), 2-OHNa; fluorene metabolites: 2- hydroxyfluorene (2-OHFlu), 9-OHFlu; phenanthrene metabolites: 1-hydroxyphenanthrene (1-OHPh), 2-OHPh, 3-OHPh, 4-OHPh, 9-OHPh; chrysene metabolite: 6-hydroxychrysene (6-OHChr); and benzo[a]pyrene metabolite:	The elevated total concentration of all PAH metabolites (ΣOH-PAHs) was associated with decreased LF and LF/HF (*P* for trend = 0.005 and < 0.0001, respectively)	08
						3-hydroxybenzo[a]pyrene (3-OHBaP)] were measured by gas chromatography–mass spectrometry (GC/MS, Agilent 6890N+5975B, Agilent Technologies Inc., Santa Clara, CA, USA).		
Trasande et al. (35)	Polycyclic aromatic hydrocarbons, brachial artery distensibility, and blood pressure among children residing near an oil refinery	Cross-sectional	184 adolescent males	Jeddah, Saudi Arabia	Systolic and diastolic BP	High performance liquid chromatography (HPLC) system was used to measure 1-, 2-, 3-, 4-hydroxyphenanthrene and 1-hydroxypyrene.	Systolic (0.47 SD units, *P* = 0.006) and diastolic (0.53 SD units, *P* < 0.001), BP Z-scores were highest at the maximum PAH, with a 4.36-fold increase in prehypertension (*P* = 0.001)	07
Shiue et al. (36)	Are urinary polyaromatic hydrocarbons associated with adult hypertension, heart attack, and cancer? USA NHANES, 2011–2012	Time-series study	5560 adults	US	Hypertension	Gas chromatography tandem mass spectrometry (GC-MS/ MS) was used to measure 2-hydroxyfluorene, 3-hydroxyfluorene, 9-hydroxyfluorene, 1-hydroxyphenanthrene, 2-hydroxyphenanthrene, 3-hydroxyphenanthrene, 1-hydroxypyrene, 1-hydroxynapthalene, 2-hydroxynapthalene, 4-hydroxyphenanthrene.	Urinary 4-hydroxyphenantheren was associated with hypertension (OR: 1.33, 95% CI: 1.00–1.76, *P* = 0.048, PAR: 5.1%), urinary 1-hydroxypyrene was significantly associated with heart attack (OR:1.47, 95% CI: 1.05–2.06, *P* = 0.027, PAR: 1.7%), and urinary 2-hydroxynaphthalene (2-naphthol) was associated with cancer (OR: 1.46, 95% CI: 1,12–1.90, *P* = 0.008, PAR: 3.9%)	09
Bangia et al. (37)	A cross-sectional analysis of polycyclic aromatic hydrocarbons and diesel particulate matter exposures and hypertension among individuals of Mexican origin	Cross-sectional	11,218 individuals	Texas	Hypertension	The National Emissions Inventory is used by NATA to provide countrywide estimates of ambient air levels of hazardous air pollutants. In NATA, 16 PAHs are classified as Polycyclic Organic Matter (PAHPOM) a wide category of chemicals.	A positive association between PAHs and hypertension (medium exposure, AOR = 1.09, 95% CI: 0.088–1.36; high exposure, OR = 1.40, 95% CI: 1.01–1.94)	10
Rajbar et al. (38)	Urinary Biomarkers of Polycyclic Aromatic Hydrocarbons Are Associated with Cardiometabolic Health Risk	Retrospective cohort 2001–2008	4,765 adult participants	US	Cardio-metabolic health risk	Capillary gas chromatography with high-resolution mass spectrometry was used to quantify the metabolites of interest. A total of eight hydroxylated urinary PAH metabolites were observed including 1-hydroxynaphthalene (1-naphthalene), 2-hydroxynaphthalene (2-naphthalene), 2-hydroxyfluorene (2-fluorene), 3-hydroxyfluorene (3-fluorene), 1-hydroxyphenanthrene (1-phenanthrene), 2-hydroxyphenanthrene (2-phenanthrene), 3-hydroxyphenanthrene (3-phenanthrene), and 1-hydroxypyrene (1-pyrene).	PAH is related to obesity and the expression of a number of obesity-related cardio-metabolic health risk factors (P < 0.05)	09
Jacobs et al. (39)	Acute changes in pulse pressure in relation to constituents of particulate air pollution in elderly persons.	Panel study	88 non-smoking persons	Antwerp, Belgium	Systolic and diastolic BP	The measured oxy-PAHs included phenanthrene-9, 10-dione, chrysene-5,6-dione, benzo[a]pyrene-4,5-dione, benzo[a]pyrene-1,6- dione, benzo[a]pyrene-3,6-dione, benzo[a]pyrene-6,12-dione, 4-oxa-benzo[def]- chrysene-5-one, 7H-benzo[de]anthracene-7-one, pyrene-1-carboxaldehyde, benz- [a]anthracene-7,12-dione, napthacene-5,12-dione.	Each PAHs increase of 20.8 μg/m ≥ in 24-h mean outdoor PM (2.5) was associated with an increase in pulse pressure of 4.0 mmHg (95% CI: 1.8–6.2), in a person taking antihypertension medication (*n* = 57), but not in person not using antihypertensive medication (*n* = 31) (P for interaction: 0.02)	10
Clark et al. (17)	Exposure to polycyclic aromatic hydrocarbons and serum inflammatory markers of cardiovascular disease	Time-series study	3,219 participants	US	CVD	Urinary concentrations of PAH metabolites were measured by means of capillary gas chromatography combined with high-resolution mass spectroscopy.	There is no significant relationship between PAH exposure and CVD disease	09
Everett et al. (21)	Association of urinary polycyclic aromatic hydrocarbons and serum C-reactive protein	Time-series study	999 participants	US	Inflammation and atherosclerosis	Nine monohydroxy polycyclic aromatic hydrocarbons were measured in urine samples using enzymatic deconjugation, followed by automated solid-phase extraction and quantified by gas chromatography/isotope dilution high-resolution spectrometry.	OH-PAHs were classified as low, medium, and high. Low OH-PAH was 2-hydroxyphenantherene ≤ 48 ng/g creatinine and 9-hydroxyfluorene ≤ 160 ng/g creatinine. High OH-PAH was 2-hydroxyphenanthrene > 148 ng/g creatinine or 9-hydroxyfluorene > 749 ng/g creatinine	10
Xu et al. (5)	Studying associations between urinary metabolites of PAHs and cardiovascular diseases in the United States	Time-series study	13,156 people	US	Cardiovascular disease	Gas chromatography/ isotope dilution high-resolution mass spectrometry (GC/IDHRMS) was used to measure the OH-PAHs metabolites in urine samples. These OH-PAHs studied are: naphthalene metabolites [i.e., 1- hydroxynaphthalene (1-NAP) and 2-hydroxynaphthalene (2- NAP)]; fluorene metabolites [i.e., 2-hydroxyfluorene (2-FLUO), and 3-hydroxyfluorene (3-FLUO)]; phenanthrene metabolites [i.e., 1- hydroxyphenanthrene (1-PHEN), 2-hydroxyphenanthrene (2- PHEN), and 3-hydroxyphenanthrene (3-PHEN)]; and pyrene metabolite [i.e., 1-hydroxypyrene (1-PYR)].	PAH was significantly associated with self-reported CVD. Patients within the middle and highest tertiles had higher self-reported CVD (the second tertile: AOR = 1.29, 95% CI: 0.97–1.72; the third tertile: AOR = 1.45, 95% CI: 1.01–2.07; *P* for trend = 0.04)	09
Burstyn et al. (3)	Polycyclic aromatic hydrocarbons and fatal ischemic heart disease	Retrospective cohort 1953–2000	12,367 male asphalt workers	Denmark, Finland, France, Germany, Israel, Netherlands, and Norway	Fatal ischemic heart disease	Benzo(a)pyrene were measured.	There is a positive relationship between benzo(a)pyrene exposure of 273 ng/m3 or higher, for which the relative risk was 1.64 (95% CI: 1.13–2.38)	08

*ASCVD, Atherosclerotic cardiovascular disease; PAHs, Polycyclic aromatic hydrocarbons; HRV, Heart rate variability; CHDs, Congenital heart diseases; CVDs, Cardiovascular disease; Electrocardiogram, ECG; CC16, Club cell secretory protein; OR, Odds ratio; AOR, Adjusted OR; CI, Confidence interval; HF, High frequency; LF, Low frequency; OR, Odds ratio; MPV, mean platelet volume; BP, Blood pressure; SD, Standard deviation; COEs, Coke oven emissions; PM, Particulate matter*.

### Characteristics of Included Studies

The systemic review included 20 studies with a variety of geographical studies. The most common research category among the nominated studies were time-series studies (5, 17, 21, 36, 40), followed by retrospective cohort (3, 28, 33, 38, 41, 42), cross-sectional (19, 26, 27, 30, 35, 37, 43), quasi-experimental (34), panel (39) and case-control (29) studies. The maximum number of individuals was 13,156 individuals, as described in one time-series study (5), whereas the lowest number of participants was described as 88 individuals in a panel study (39). Most of the studies (5, 17, 21, 33, 34, 36–38, 40, 41, 44) were conducted in the United States, while others were presented in various geographical countries around the world, such as Denmark, Germany, Finland, Netherlands, France, Norway (3), China (19, 25, 27–30, 45), Korea (26), Sweden (31), Saudi Arabia (35), and Belgium (39). In most of the published studies, the result (outcome) was reported as CVDs (5, 17, 25, 33), including as fatal ischemic heart disease (3), heart rate variability (19, 27, 34), atherosclerotic cardiovascular disease (28, 46), congenital heart disease (29), cardiovascular events (47), inflammation and atherosclerosis (21), and cardio-metabolic heart rate (38). Though the outcome was described as blood pressure (BP) with the titles of hypertension (26, 36, 37) and systolic and diastolic BP (35, 39) in three of the studies, and as obesity (38, 41) in one of them.

### Association of PAH Exposure With Cardiovascular Diseases

A total of twenty studies comprised in this review, eight studies evaluated the association between PAH exposure and CVDs (9, 11, 13, 14, 16, 18, 48), four articles observed this relationship with BP (33, 49–51), two observed association between atherosclerotic cardiovascular disease and PAH (28, 46), one Congenital heart disease (29), Cardiovascular events (47) and two with obesity (13, 17). Table 1 presents a list of key findings of these research articles. The majority of evidence has shown a substantial positive relationship between exposures to PAH and the risk of CVDs (5, 19, 33, 34, 38). However, the findings of Clark et al. (17) indicated the contrary, claiming that there was no substantial association between exposures of PAH and CVDs risk. Furthermore, a positive relationship was testified between PAH exposure and hypertension (21, 35–37, 39), as well as between PAH exposure and obesity (38, 41) in some of the studies.

## Discussion

In this systematic review, we evaluated the association between exposure to PAH and cardiovascular diseases. The results indicated that exposure of PAH and risk of CVDs were significantly positively associated. PAH-rich sources are recognized risk factors affecting the human cardiovascular system, including cigarette smoke (3), cooking smoke, and exhaust smokes (52–54). According to studies conducted in this area, in PHA-contaminated settings, people with cardiometabolic risk factors are more vulnerable; the elderly (55), as well as people with diabetes (56), obesity (57), heart disease (58), and high systemic inflammation (59), are more affected. A cross-sectional study (5) found that the prevalence of self-reported CVDs is positively associated with PAH exposure. Nevertheless, another study (17) reported no significant correlation between PAH exposure and inflammatory CVDs; however, this study did not answer the potential underlying explanations for their results being adequately supported. In addition, another study showed that PAH biomarkers were linked with elevated diastolic blood pressure, indicating that PAH exposure is a contributing element in the development of CVD (31). Wuhan-Zhuhi (WHZH) cohort study, which included 2,715 individuals aged 30–74 years, observed a strong positive association between urinary OH-PAHs levels with the 10-year ASCVD risk (28). A U.S. population-based study, merged 2001–02, 2003–04, 2005–06, 2007–08, and 2009–10 data cycles, included 7,301 total participants, found a positive association between PAH exposure and CVD (33).

Furthermore, studies demonstrated that urinary low molecular weight OH-PAHs were linked with raised prevalence of 10-years ASCVD in a Chinese population (25). Urinary 8-oxodG was significantly linked with PAH exposure and 10-year risk of ASCVD (25, 28). In addition, an association was observed between PAH exposures and hypertension in people (26). In the United States, there was a positive dose-response association was noticed for urinary 2-naphthol and 2-hydroxyphenanthrene and hypertension (26, 38). A study evaluated those greater maternal levels of PAHs exposure during pregnancy may be linked to an elevated prevalence of fetal CHDs and CHDs subtypes (29). Earlier studies in laboratory model systems have indicated that prenatal PAH exposure is linked with CHDs (60, 61).

Moreover, the findings indicated that sensitivity to PAH be associated with raised BP significantly. Accordingly, systolic and diastolic BP has been reported to be greater in school students near the oil factories and in those who are exposed to significant amounts of this substance than in schools outside that area (35). Another research found that with rising age, living in high-traffic areas, and body mass index, the prevalence of hypertension increases (37). Similarly, reports conducted on people with raised cholesterol, myocardial infarction history, or diabetes, and those with physical disabilities, presented an increased prevalence of hypertension due to exposure to PAH. A positive association is also documented between exposure to PAH and the level of BP (39). Experimental studies have shown that exposure to organic compounds containing PAH may lead to elevated arterial BP (50).

The pathways that underlying the substantial link between PAH exposure and CVD are yet unclear. Detoxification occurs in response to PAH exposure, resulting in the production of extremely reactive metabolites that can interact with DNA (62). PAH exposure was reported to have dose-dependent effects on plaque development in animals (63). Pre-clinical investigations have also shown that PAHs may cause atherosclerosis by inducing an inflammatory response that results in an enhanced infiltration of pro-inflammatory cells into plaques (9). Inflammation has been recognized as a risk factor for the development of CVD (64, 65). There was an association observed between PAH and inflammation, according to population studies (15, 17). Recent research also found an association between PAHs and a variety of obesity-related cardio-metabolic risk factors (38). PAH exposure is positively linked with systemic inflammation and oxidative stress in the pathogenesis of atherosclerosis, according to accumulating evidence (15, 66). The expression of pro-inflammatory cytokines in carotid plaques was triggered by DNA adducts in animal arteries (9). Furthermore, exposure to PAHs exacerbated atherosclerosis in HepG2 cells via the activation of p53 and causing down-regulation of the liver X receptor-mediated genes (67).

Moreover, the evidence suggested that PAH exposure was linked with obesity and cardio-metabolic risk factors (38, 68). In children and non-diabetic adults, urinary PAHs metabolites were positively associated with biomarkers of cardio-metabolic risk (such as BMI and WC), IR, and improved prevalence of metabolic syndrome (68, 69). PAHs may be found in all internal organs of humans, particularly in adipose tissue, due to their high lipophilicity. Parent PAHs and their metabolites have been linked to estrogen receptor activation and thyroid receptor inhibition (70). They modify fat cell metabolism, resulting in a rise in weight and fat mass, which is linked to the alternation of metabolic homeostasis and IR during the development of adipose tissue via elevated gene expression, adiponectin, and reduced DNA methylation of peroxisome proliferator-activated receptor γ (71). Toxicological research found a substantial link between low-dose PAH exposure and adiposity (72). According to the research, adipose tissue can act as a reservoir for inflammatory cytokines and numerous chemicals, including PAHs, and can play a crucial role in the advancement of atherosclerosis and carcinogenesis in both acute and chronic states (73). PAH exposure has been linked to metabolic outcomes of amino acids, purine, lipid, and glucuronic acid along with human oxidative stress state (74). One of the most powerful PAH carcinogens, benzo[a]pyrene, affects phase I and phase II enzyme activity via increasing AhR-dependent gene expression, raises oxidative stress, and hence causes cellular dysfunction, including muscle protein breakdown and adipocyte differentiation (75). Furthermore, PAH exposure causes an inflammatory response, the production of PAH-DNA adducts, and the suppression of DNA repair in the heart tissue, with larger PAH-DNA adducts in smokers' hearts and other tissues than non-smokers' (76). Moreover, oxidative stress-related mitochondrial DNA damage has been linked to the development of several atherogenic pathways (73). Adiposity has been linked to a greater risk of CVDs as an independent health risk factor (77, 78).

PAHs are prevalent environmental contaminants across the world, and chronic CVDs, including congestive heart failure, coronary artery disease, angina, heart failure, and stroke are the main globally public health concerns, the findings of our study have significant global health consequences. The positive exposure-response relationship between PAH exposure and the increased prevalence of CVDs from our study indicates that regulating atmospheric PAHs may be an efficient strategy to lower the increased risk of CVDs and prevent cardiovascular disease.

### Limitations

Our study has some noteworthy limitations. First, the most notable was the absence of sufficient evidence to address a number of the review questions. Our ability to synthesize the available evidence was also limited by the variation in design, methodology, samples, analysis, and presentation of results of the included studies. However, given the limited number of studies available in the area, and the aim of our study to provide an overview of the literature, it was important to include all available evidence regardless of design. Second, our study is an only systematic review, our study is limited only to the selected database source and English-language publications. Third, although this analysis provides evidence for the harmful effects of PAH, we are unable to comment on the potential of interventions, such as PAH pathways and their effects on a cellular and molecular level. Fourth, we did not do a meta-analysis and have an adequate number of studies to pool to assess the association.

### Prospects

This systematic review may be useful for future investigation and development of therapeutic strategies for the removal of PAHs from the environment. Most studies are from developed countries and it is important to highlights the impacts of PAH pollution in developing countries and identify the existing gap in human health and environmental exposures. In this area, longitudinal research types with long-term follow-up are important. However, our study provides important data from which future practice-changing prospective trials can be designed.

## Conclusions

This systematic review examined the relationship of PAH exposure with CVDs and CVD-related risk factors by searching several digital databases. After a comprehensive literature searches and summarizing findings from 20 articles, the authors concluded that a positive relationship was observed between PAH exposure and CVD risks. Overall, epidemiological results in both occupational and the general population recommend potential relationships between environmental PAH exposure and CVDs, and other well CVDs risk factors.

### Recommendations for Health and Environmental Protection

- Due to the widespread presence of PAHs in the environment and their toxicological significance, the assessment of exposure to PAHs is significant.
- The biological impact should be precisely monitored in terms of the total PAHs intake into the body via respiratory, dermal, and gastrointestinal routes.
- PAH exposure in occupational settings should be eliminated, or reduced due to its toxic effects
- Public awareness and education should be strengthened about the causes and health effects of PAH exposure.
- Air pollution should be documented throughout the year and not only just seasonally.

## Data Availability Statement

Publicly available datasets were analyzed in this study. This data can be found here: Pubmed, Scopus and Web of Science.

## Author Contributions

All authors contributed to conception and design, interpretation of the data, critical revision of the manuscript, and approval of the final version to be published.

## Conflict of Interest

The authors declare that the research was conducted in the absence of any commercial or financial relationships that could be construed as a potential conflict of interest.

## Publisher's Note

All claims expressed in this article are solely those of the authors and do not necessarily represent those of their affiliated organizations, or those of the publisher, the editors and the reviewers. Any product that may be evaluated in this article, or claim that may be made by its manufacturer, is not guaranteed or endorsed by the publisher.
