# Supplementary material for: Relationship Between Polycyclic Aromatic Hydrocarbons and Cardiovascular Diseases: A Systematic Review
Source: Front Public Health. 2021 Dec 7;9:763706. doi: 10.3389/fpubh.2021.763706 (PMC8688693; doi:10.3389/fpubh.2021.763706)
Supplement: Supplementary file 1 [file Table_1.DOCX]

**Supplementary table** 1: Search Strategies

| Sources | Search strategies |
| --- | --- |
| PubMed/MEDLINE | (((((((Polycyclic Aromatic Hydrocarbon) OR (PAH)) OR (Polynuclear Aromatic Hydrocarbon)) OR (Polyaromatic Hydrocarbon)) OR (Aromatic Polycyclic Hydrocarbon)) OR (Polycyclic Aromatic Compound)) AND ((((((((((((((((((heart diseases) OR (vascular diseases)) OR (death, sudden)) OR (heart disease)) OR (stroke)) OR (cerebrovascular accident)) OR (sudden death)) OR (cardiac arrest)) OR (cardiovascular disease)) OR (coronary artery disease)) OR (heart failure)) OR (cardiovascular mortality)) OR (coronary death)) OR (CHD)) OR (CVD)) OR (cardiac death)) OR (myocardial infarction)) OR (angina))) AND ((((((Randomized Controlled Trial [Publication Type]) OR (Controlled Clinical Trial [Publication Type])) OR (Randomized [Publication Type])) OR (Placebo[Title/Abstract])) OR (randomly[Title/Abstract])) OR (trial[Title/Abstract])) |
| Web of Science | (((((((TS=(Polycyclic Aromatic Hydrocarbon)) OR TS=(PAH)) OR TS=(Polynuclear Aromatic Hydrocarbon)) OR TS=(Polyaromatic Hydrocarbon)) OR TS=(Aromatic Polycyclic Hydrocarbon)) OR TS=(Polycyclic Aromatic Compound)) AND TS=((((((((((((((((((TS=(heart diseases)) OR TS=(vascular diseases)) OR TS=(death, sudden)) OR TS=(heart disease)) OR TS=(stroke)) OR TS=(cerebrovascular accident)) OR TS=(sudden death)) OR TS=(cardiac arrest)) OR TS=(cardiovascular disease)) OR TS=(coronary artery disease)) OR TS=(heart failure)) OR TS=(cardiovascular mortality)) OR TS=(coronary death)) OR TS=(CHD)) OR TS=(CVD)) OR TS=(cardiac death)) OR TS=(myocardial infarction)) OR TS=(angina))) AND TS=((((((TS=(Randomized Controlled Trial)) OR TS=(Controlled Clinical Trial)) OR TS=(Randomized)) OR TS=(Placebo)) OR TS=(randomly)) OR TS=(trial)) |

Polycyclic Aromatic Hydrocarbon

PAH

Polynuclear Aromatic Hydrocarbon

Polyaromatic Hydrocarbon

Aromatic Polycyclic Hydrocarbon

Polycyclic Aromatic Compound”

Randomized Controlled Trial [Publication Type]

Controlled Clinical Trial [Publication Type]

Randomized [Publication Type]

Placebo[Title/Abstract]

randomly[Title/Abstract]

trial[Title/Abstract]

heart diseases

vascular diseases

death, sudden

heart disease

stroke

cerebrovascular accident

sudden death

cardiac arrest

cardiovascular disease

coronary artery disease

heart failure

cardiovascular mortality

coronary death

CHD

CVD

cardiac death

myocardial infarction

angina

#1

(((((Polycyclic Aromatic Hydrocarbon) OR (PAH)) OR (Polynuclear Aromatic Hydrocarbon)) OR (Polyaromatic Hydrocarbon)) OR (Aromatic Polycyclic Hydrocarbon)) OR (Polycyclic Aromatic Compound)

#2

(((((Randomized Controlled Trial [Publication Type]) OR (Controlled Clinical Trial [Publication Type])) OR (Randomized [Publication Type])) OR (Placebo[Title/Abstract])) OR (randomly[Title/Abstract])) OR (trial[Title/Abstract])

#3

(((((((((((((((((heart diseases) OR (vascular diseases)) OR (death, sudden)) OR (heart disease)) OR (stroke)) OR (cerebrovascular accident)) OR (sudden death)) OR (cardiac arrest)) OR (cardiovascular disease)) OR (coronary artery disease)) OR (heart failure)) OR (cardiovascular mortality)) OR (coronary death)) OR (CHD)) OR (CVD)) OR (cardiac death)) OR (myocardial infarction)) OR (angina)

##1

(((((TS=(Polycyclic Aromatic Hydrocarbon)) OR TS=(PAH)) OR TS=(Polynuclear Aromatic Hydrocarbon)) OR TS=(Polyaromatic Hydrocarbon)) OR TS=(Aromatic Polycyclic Hydrocarbon)) OR TS=(Polycyclic Aromatic Compound)

##2

(((((((((((((((((TS=(heart diseases)) OR TS=(vascular diseases)) OR TS=(death, sudden)) OR TS=(heart disease)) OR TS=(stroke)) OR TS=(cerebrovascular accident)) OR TS=(sudden death)) OR TS=(cardiac arrest)) OR TS=(cardiovascular disease)) OR TS=(coronary artery disease)) OR TS=(heart failure)) OR TS=(cardiovascular mortality)) OR TS=(coronary death)) OR TS=(CHD)) OR TS=(CVD)) OR TS=(cardiac death)) OR TS=(myocardial infarction)) OR TS=(angina)

##3

(((((TS=(Randomized Controlled Trial)) OR TS=(Controlled Clinical Trial)) OR TS=(Randomized)) OR TS=(Placebo)) OR TS=(randomly)) OR TS=(trial)
